# Psychache, Coping Strategies, and Therapeutic Approaches Among Palestinian University Students During War

**DOI:** 10.1192/j.eurpsy.2026.11648

**Published:** 2026-07-31

**Authors:** H. K. Yacoub, S. Sabbah, Z. Yacoub, R. Yacoub, R. R. Holden

**Affiliations:** 1 Faculty of Medicine; 2 Psychology department, Al Quds University, Abu Dis, West Bank and Gaza; 3 Department of Psychology, Queen’s University, Ontario, Canada

## Abstract

**Introduction:**

The prolonged Gaza-Israeli war has profoundly impacted the psychological well-being of Palestinian university students, manifesting in elevated levels of psychache - an intense psychological pain marked by despair, humiliation, and helplessness. Despite its established link to suicidal ideation, the relationship between psychache and coping strategies during wartime remains underexplored. Palestinian students are uniquely vulnerable, living in a context of ongoing trauma, instability, and restricted access to mental health resources. The continuous exposure to war images, financial crisis, and fear for loved ones may disrupt their sense of security and increase their vulnerability to psychache, increasing the urgency for context-specific mental health assessment and intervention.

**Objectives:**

This study aims to assess the prevalence and severity of psychache among Palestinian university students in the West Bank during the Gaza-Israeli war, and to identify the coping strategies employed.

**Methods:**

A cross-sectional study was conducted with 556 participants from various universities in the West Bank. Data were collected via online questionnaires, including the Psychache Scale, Orbach and Mikulincer Mental Pain Scale, and Coping Strategies Questionnaire. Descriptive statistics, t-tests, and ANOVA were used to analyze the data.

**Results:**

Psychache Associations

- Significantly related to: marital status, year of study, level, and specialization (p < 0.05)
- Not related to: gender, place of residence (p > 0.05)

**Table 1.** Psychache levels among the sample Table 1. Long description.

Scale	Mean	SD	High Psychache	Moderate	Low
Psychache Scale	31.68	10.91	10.1%	41.2%	48.7%
Orbach & Mikulincer Scale	20.43	7.05	13.5%	38.8%	47.7%

**Table 2.** Coping Strategies (Means and SD) Table 2. Long description.

Strategy	Mean	SD
Catastrophizing	13.82	5.89
Diversion	19.34	6.36
Reinterpreting	16.52	5.92
Cognitive Coping	15.88	5.32

**Table 3.** Correlations Between Variables Table 3. Long description.

Variables	Psychache	Orbach	Catastro.	Diversion	Reinterp.	Cog. Coping
Psychache	—	0.71**	0.69**	0.32**	0.38**	0.26**
Orbach & Mikulincer Scale		—	0.71**	0.26**	0.46**	0.23**

**Image 1:**
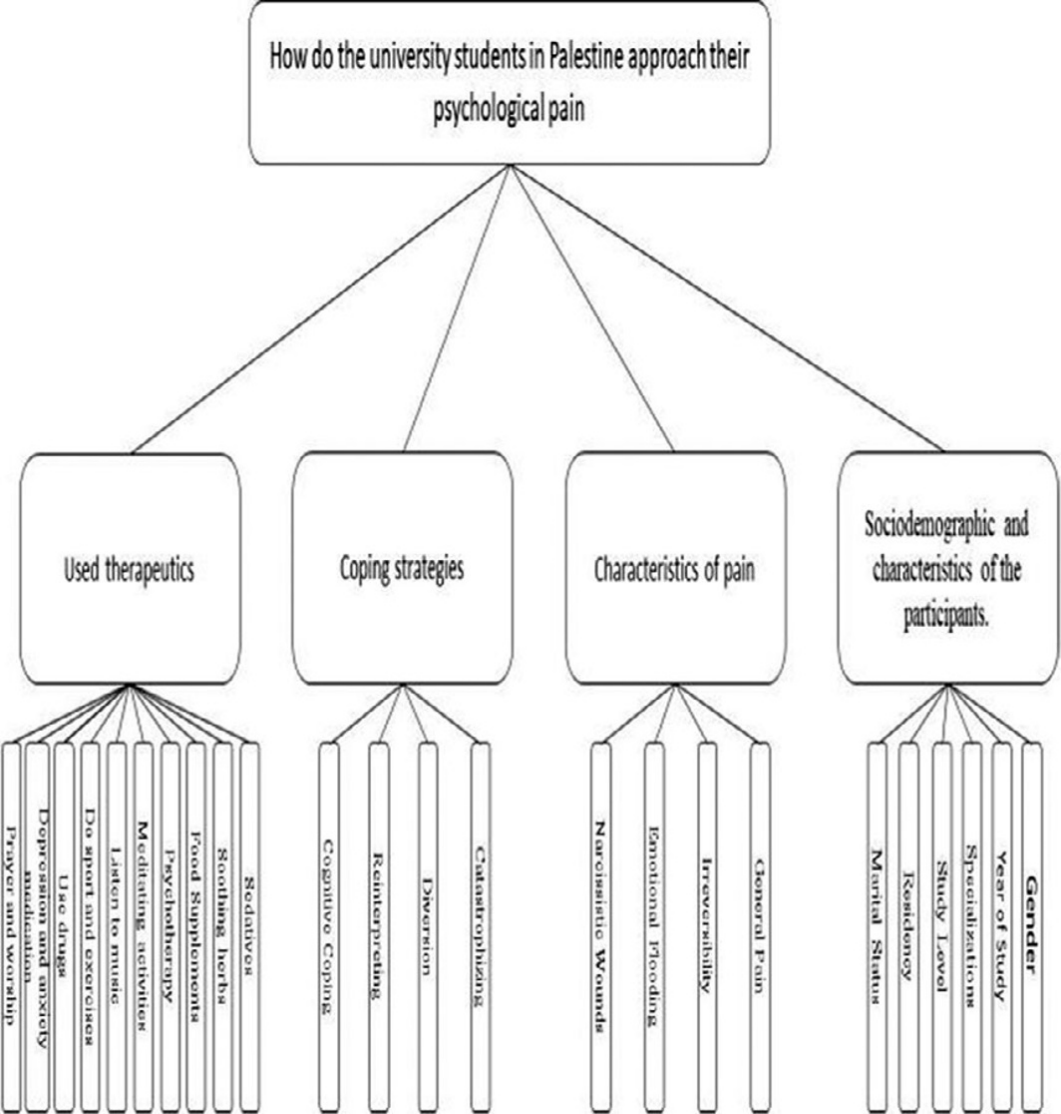
Image 1: Long description.

**Conclusions:**

The study underscores the critical need for targeted mental health interventions among university students during war situations. Enhanced coping strategies and mental health support tailored to students’ demographics and psychosocial context are essential for mitigating the impact of war on psychological well-being. Religious and cultural factors must be considered in mental health interventions, especially in societies like Palestine where faith-based coping is widely practiced. This study contributes to a growing body of evidence calling for culturally adapted mental health frameworks in conflict zones.

**Disclosure of Interest:**

None Declared

## Long descriptions

### Table 1. Long description

The table consists of six columns: Scale, Mean, S D, High Psychache, Moderate, and Low.

* The first data row for the Psychache Scale shows a Mean of 31.68, an S D of 10.91, with 10.1 percent High Psychache, 41.2 percent Moderate, and 48.7 percent Low.

* The second data row for the Orbach and Mikulincer Scale shows a Mean of 20.43, an S D of 7.05, with 13.5 percent High Psychache, 38.8 percent Moderate, and 47.7 percent Low.

Navigate back to Table 1..

### Table 2. Long description

The table consists of three columns labeled Strategy, Mean, and S D. It contains four rows of data.

* Row 1: Catastrophizing has a Mean of 13.82 and an S D of 5.89.

* Row 2: Diversion has a Mean of 19.34 and an S D of 6.36.

* Row 3: Reinterpreting has a Mean of 16.52 and an S D of 5.92.

* Row 4: Cognitive Coping has a Mean of 15.88 and an S D of 5.32.

Navigate back to Table 2..

### Table 3. Long description

The table consists of seven columns and two primary data rows. The columns are labeled: Variables, Psychache, Orbach, Catastro. (Catastrophizing), Diversion, Reinterp. (Reinterpretation), and Cog. Coping (Cognitive Coping).

Row 1: Psychache. The correlation with itself is marked by a dash. It shows positive correlations with Orbach at 0.71, Catastro. at 0.69, Diversion at 0.32, Reinterp. at 0.38, and Cog. Coping at 0.26. All values are marked with double asterisks indicating statistical significance.

Row 2: Orbach and Mikulincer Scale. The correlation with Psychache is blank (as it was covered in Row 1). The correlation with itself is marked by a dash. It shows positive correlations with Catastro. at 0.71, Diversion at 0.26, Reinterp. at 0.46, and Cog. Coping at 0.23. All values are marked with double asterisks.

Navigate back to Table 3..

### Image 1: Long description

A hierarchical flowchart begins with a top central box containing the question: How do the university students in Palestine approach their psychological pain. Four lines descend from this box to four primary category boxes arranged horizontally.

1. Used therapeutics (far left): This category branches into ten vertical sub-items: Prayer and worship, Depression and anxiety medication, Use drugs, Do sport and exercises, Listen to music, Meditating activities, Psychotherapy, Food Supplements, Soothing herbs, and Sedatives.

2. Coping strategies (middle-left): This category branches into four vertical sub-items: Cognitive Coping, Reinterpreting, Diversion, and Catastrophizing.

3. Characteristics of pain (middle-right): This category branches into four vertical sub-items: Narcissistic Wounds, Emotional Flooding, Irreversibility, and General Pain.

4. Sociodemographic and characteristics of the participants (far right): This category branches into six vertical sub-items: Marital Status, Residency, Study Level, Specializations, Year of Study, and Gender.

Navigate back to Image 1:.

